# Ten simple rules for writing statistical book reviews

**DOI:** 10.1371/journal.pcbi.1006562

**Published:** 2019-01-24

**Authors:** Christopher J. Lortie

**Affiliations:** 1 Department of Biology, York University, Toronto, ON, Canada; 2 The National Center for Ecological Analysis and Synthesis, UCSB, Santa Barbara, California, United States of America; Dassault Systemes BIOVIA, UNITED STATES

## Abstract

Statistical books can provide deep insights into statistics and software. There are, however, many resources available to the practitioner. Book reviews have the capacity to function as a critical mechanism for the learner to assess the merits of engaging in part, in full, or at all with a book. The “ten simple rules” format, pioneered in computational biology, was applied here to writing effective book reviews for statistics because of the wide breadth of offerings in this domain, including topical introductions, computational solutions, and theory. Learning by doing is a popular paradigm in statistics and computation, but there is still a niche for books in the pedagogy of self-taught and instruction-based learning. Primarily, these rules ensure that book reviews function as a form of short syntheses to inform and guide readers in deciding to use a specific book relative to other options for resolving statistical challenges.

## Author summary

Book reviews are a useful tool to inform learners in statistics and computational biology. As an ecologist, I teach biostatistics and use many resources in the analysis and coding of research data. In-depth texts can provide a critical resource, but well-written reviews can faciliate the decision to use a specific book.

## Introduction

Extensive resources now support the statistical programmer and analyst. The learner, reader, and general problem solver is thus faced with a choice of how to learn what is needed [1,2]. This brief synthesis is not intended to be a comment or criticism on the pedagogy associated with successfully acquiring statistical and coding expertise, but there is evidence suggesting that up to 80% of coders do not read books to learn how to code [6]. This seems like an unfortunate statistic, but the philosophy of “learning statistics by doing statistics” is not without merit and can be a viable approach to both introductory and expert learners alike [4]. Nonetheless, R, Python, SAS, and MATLAB/C++ are quite literally deep languages that need to be mastered. Fluency in a written or spoken language conveys reason and semantics [5]; statistical reasoning [4] with a corresponding representation of the associated mathematics [3] can likely be secured by both doing and reading [7]. Different problems and topics can also require the statistical programmer to embrace a diversity of resources to illuminate a solution, and the depth required must be defined by the prior knowledge of an individual and nature of the challenge.

Many statistical texts can be a significant time commitment, and open electronic resources are abundant. The decision to read a statistical programming book is not necessarily trivial. Short syntheses, i.e., a review, of the relative merits of a specific resource can provide a critical decision tool to the potential reader. The “ten simple simple rules” paper format was pioneered by Philip Bourne in *PLOS Computational Biology* [14], and it has proliferated to nearly 100 papers, all functioning as a succinct, unique form of synthesis in itself [8]. Sometimes extensive resources are summarized that support how to describe a focused process or get a task done in many domains of the scientific endeavor [11]. Of these “ten simple rules” papers, there have been three that address the review process, including how to be an effective referee [9], how to write a literature review [12], and how to write a reply paper [10]. Many of these rules certainly support improvements in how to write a review of statistical books and should be consulted. Yet, book reviews in the *Journal of Statistical Software*, e.g., strongly suggest that the importance of this topic warrants specific treatment because these reviews can serve many functions from descriptive summary to critical analysis to a launchpad for the importance of a statistical test, approach, program, language, and/or package. All are important functions that advance statistics, but at least some of the rules here can enhance their capacity to assess merit and need for the end practitioner. (Appropriately) defend books. Write reviews. Use reviews. Book reviews that effectively support the decision process for better statistical reasoning are needed. These rules promote this paradigm shift.

## Rules

### Rule 1: Introduce the topic

The book title is an excellent starting point for the reader to assess whether this is the resource for her but not the only mechanism. The book cover or sleeve synopsis and publisher description can also fail to capture the whole story, and some statistical treatises, both introductory and advanced, necessarily invoke related principles and topics [13]. As the objective expert of that specific text, an introduction to the necessity, scope, depth, and breadth of the topic in general can inform the reader on the challenges and solutions, including types of data or domains of inquiry that this field examines. Place the work within the span of the literature with a brief explanation of the area in which it is embedded. The goal of the first rule is therefore to ensure that the reader is in right place—conceptually, at least.

### Rule 2: State assumed audience (i.e., expertise-level for the reader)

Most technical book reviews state the level of expertise required by the reader. This is a critical form of synthesis that should be mentioned, even in brief, in a book review for statistics. The most typical categories range from introductory to advanced, with relatively higher-level offering described by “graduate student” and beyond as the reader. If the text is a blend of theory and practice with significant programming, the review should further explain the relative expertise needed for each and whether both dimensions are aligned in the assumed relative audience. Book reviews can also take the opportunity here to frame this assessment by the expertise of the referee (i.e., it is sometimes useful to know if the book reviewer is a statistician, a programmer, or a domain-specific end-user) or by the intended use of the text, such as primer, guide, in-depth treatise, or textbook appropriate for instruction at a given level.

### Rule 3: Explain different editions

If more than one edition exists, it is useful to describe the revisions to the more recent version of a book. Professional and teaching textbooks can be relatively expensive, and a critical assessment of value can provide instructors with the merits associated with potentially higher costs to students of purchasing a newer text. At minimum, a list of additions will facilitate a more informed choice for the reader and instructor, and mention of case studies, updates to code and data sets, and addition of supplements are all important criteria for the choice to learn or seek solutions from a specific edition.

### Rule 4: Describe the structure of chapters and associated pedagogy

Organization of the content matters to all learning [15], and content provides context [16]. The structure of statistical and programming texts can vary significantly. The length and complexity of chapters, use of headings and subsections within chapters, and end-of-chapter summaries are not always needed but often do no harm. Case studies, appendices, data sets, and location of supplements and supporting materials should be described. Contemporary texts in statistics are often a hybrid of print and electronic resource materials, and description of the extent that a text functions in this capacity can influence the decision by the reader based on her preferred modality of learning and the rationale for exploring this topic. This is also a good place to mention the different formats of the book (if available in print and online). As the reviewer, use parsimony and caution in deciding what level of detail to describe for the structural elements of a book—focus only on those elements that promoted the most effective learning.

### Rule 5: Summarize content

These are the results, so to speak, similar to a conventional scientific publication or study report. The description should be brief, topological, and highlight the most substantive elements of the book. This component of the book review need not be unduly critical but should provide an overview of the what the text offers. Some reviews take this description of what is done to also highlight what is done best and list the most valuable chapters to the reader. This can be a useful guide to the potential reader and a means to assess expectations from the book as a whole. If there are data sets or case studies that are revisited throughout the book or across multiple chapters, the extent that the chapters connect to one another can also be summarized. Mention whether the content of the book is serialized or if chapters can be read piecemeal.

### Rule 6: Critique readability

Readability is an intuitive concept. It is the ease that one can comprehend writing [17, 18]. Complexity in syntax, vocabulary, and sentence structure should be described in a review of a statistical book. A technical book need not be a technical challenge to read. More broadly, appeal, style, and interest are important to all but the most committed readers, and it is reasonable to assume that a book on statistics provide some sense of enthusiasm for the topic, compel the reader to think deeply, and engage one with the challenges explored. Composition is critical, particularly in long-form writing endeavors.

### Rule 7: List packages and linkages to statistical concepts

Within the R statistics community, there are now over 11,000 packages that extend the base language archived on https://cran.r-project.org. SAS Procs and libraries in Python and MATLAB are also extensive. Some statistical texts are associated with not only a single statistical program or language but with a single package or library. A review of a statistical book should thus describe the specificity of the book, explain the extent that the book relies on single solution sets, or conversely contrasts alternatives in different languages, applications, packages, and/or libraries, and frame the programming (if provided) to general statistical theory and reasoning. At times, this can be self-evident if the title of the book includes mention of the programming language or software, but the breadth of the statistics and case studies illustrated is typically not evident without review of the book. If the book is not tied to a specific computation tool in any form, then the reviewer should mention that this is the case and state that the concepts described can be applied and transferred broadly from the book.

### Rule 8: Compare the book to other resources

Compare and contrast. There is a wealth of both short- and long-form documentation available for many open coding languages used in statistics and data wrangling. There is also an extensive opportunity to seek specific solutions through numerous forums such as Stack Overflow (https://stackoverflow.com/questions/tagged/statistics), Cross Validated (https://stats.stackexchange.com), and Stack Exchange Mathematics (https://math.stackexchange.com). Online tutorials, blogs, carpentries, massive online open courses (MOOCs), and webinars often provide useful, and at times, deep-learning opportunities. A book review will certainly not comprehensively list all these options and compare and contrast to the principal subject text discussed, but if there is a significant alternative to consider, it should be mentioned. Finally, there are also other books. The reviewer should explicitly state the extent that she is contrasting to other resources, and due diligence by the reviewer suggests at the minimum a mention of the relative novelty and niche of the text in question.

### Rule 9: Comment on reading commitment and style

Reading a book is a relationship. The content, style, and perspective of the author(s) becomes a shared, internalized form of knowledge in a good book. As the reviewer, it is legitimate and useful to others to mention the extent that one enjoyed the text, connected with the writing and concepts, or struggled with certain elements (i.e., comment on the quality of the relationship with the book). A review should also mention the time that the reader should set aside to read and/or fully digest the content. If the “summarize content” rule proposed above did not mention the standout, best chapters, this is an excellent spot to describe the chapters that provided the most for your buck and should not be skipped. This is also an ideal opportunity to consider describing whether this is was a read-the-book-straight-through or piecemeal technical read for critical needs.

### Rule 10: Be professionally critical and state personal purpose

In general, it is best to be decisive in writing reviews [9]. Evaluate the capacity that the book delivers on its stated goal. Accept that you are part of the review process and likely have your own, specific purpose in reading this text. Admit this in the review by articulating the need, success of text, and decision (or not) to use the described tools, framework, or theory. Being specific and listing criteria point-by-point is useful to editors, authors, and readers [9]. Similar to the peer review process for papers, be balanced, fair, and professionally critical by mentioning both strengths and weaknesses from your perspective. Do your best to reveal implicit biases in your review.

## Summary

Reading, writing, and statistics. By putting oneself on the hook for a book to take notes and annotate or further synthesize these efforts and provide a review profoundly changes how one approaches a statistical and programming text [19, 20]. Higher education in the sciences and statistics has largely done away with book reviews and/or reports, but application and dissemination of critical thinking in statistics in the form of reviews is a learning opportunity. Capitalize on this process, particularly when using a text to solve a problem and write a review. Reviewing is a both a collegial and educational service that includes oneself as the beneficiary. The rules proposed herein for writing a book review for statistics and increasingly for the associated coding or implementation of statistics and data do not mean to imply that reading texts in this domain is a burden. On the contrary, the gratification of immersion in the structured reasoning inherent in these fields is a powerful form of literacy that merits discussion by people, for people. Recommendation algorithms certainly influence many aspects of human behavior, and a book review is a reminder to take a moment and savor the story.
